# Review: Effect of Experimental Diets on the Microbiome of Productive Animals

**DOI:** 10.3390/microorganisms11092219

**Published:** 2023-08-31

**Authors:** Rodrigo Huaiquipán, John Quiñones, Rommy Díaz, Carla Velásquez, Gastón Sepúlveda, Lidiana Velázquez, Erwin A. Paz, Daniela Tapia, David Cancino, Néstor Sepúlveda

**Affiliations:** 1 Programa de Doctorado en Ciencias Agroalimentarias y Medioambiente, Facultad de Ciencias Agropecuarias y Medioambiente, Universidad de la Frontera, Temuco 4780000, Chile; r.huaiquipan01@ufromail.cl (R.H.); c.velasquez12@ufromail.cl (C.V.); g.sepulveda10@ufromail.cl (G.S.); l.velazquez01@ufromail.cl (L.V.); daniela.tapia@ufrontera.cl (D.T.); 2 Facultad de Ciencias Agropecuarias y Medioambiente, Universidad de la Frontera, Temuco 4780000, Chile; rommy.diaz@ufrontera.cl (R.D.); david.cancino@ufrontera.cl (D.C.); 3 Centro de Tecnología e Innovación de la Carne, Universidad de La Frontera, Temuco 4780000, Chile; 4 UWA Institute of Agriculture, The University of Western Australia, Perth 6009, Australia; erwin.pazmunoz@uwa.edu.au

**Keywords:** animal production, bacteria community, gut, monogastric, new feedstuff, livestock, ruminant

## Abstract

The microorganisms that inhabit the gastrointestinal tract are responsible for multiple chains of reactions that affect their environment and modify the internal metabolism, their study receives the name of microbiome, which has become more relevant in recent years. In the near future, the challenges related to feeding are anticipated to escalate, encompassing the nutritional needs to sustain an overpopulated world. Therefore, it is expected that a better understanding of the interactions between microorganisms within the digestive tract will allow their modulation in order to provide an improvement in the immune system, feed efficiency or the promotion of nutritional characteristics in production animals, among others. In the present study, the main effects of experimental diets in production animals were described, emphasizing the diversity of the bacterial populations found in response to the diets, ordering them between polygastric and monogastric animals, and then describing the experimental diets used and their effect on the microorganisms. It is hoped that this study will help as a first general approach to the study of the role of the microbiome in production animals under different diets.

## 1. Introduction

In 2050, the population will exceed 9.7 billion inhabitants and food shortage problems will reach a critical state, aggravated by environmental problems and nutrient shortages on earth [1]. Under this context, the number of research projects related to solving food problems has increased, studies based on the microbiome being a focus of great interest, especially those related to diets in animals. To begin with, it is necessary to differentiate between the terms microbiota and microbiome, the former being defined as the “set of microorganisms present in a defined environment” [2], while microbiome can refer to the microorganisms present in a given habitat that have different physicochemical properties, interact with their environment and can confer microbiological resistance to their host [3]. Furthermore, the microbiome involves and considers interactions between bacteria, archaea, viruses, and fungi, as well as between biotic and abiotic factors involved [4,5]. The alteration of the microorganisms involved in the microbiome can cause dysbiosis, which consists of the imbalance of a healthy microbiome, where the symbiotic relationships of the microbiota are diminished, altering productivity, and causing health problems [6]. Although, studies on the microbiome have increased during the last years, they are mostly based on humans, existing a smaller number related to animals destined to food production [7].

Feed is a key element in the microbiota of animals, since it has a direct effect on microbial populations. For example, in ruminants it is proposed that polyphenols affect carbohydrate fermentation in the rumen, in addition to affecting cellular molecules such as proteins, lipids and nucleic acids [8]. At the same time, studies highlight the action of tannins to reduce fiber degradation, as well as polyphenols to help reduce methane gas production [9]. Other studies have proposed that the concentration of polyphenols affects the intestinal epithelial barrier of pigs in vitro using chestnut extracts, an important source of polyphenols, which at low concentrations can have a protective effect on intestinal epithelial cells [10]. In monogastric animals such as chickens and pigs the consumption of dietary fiber exerts significant effects on the integrity of the intestinal mucosa. Therefore, a dietary fiber deficiency leads to loss of functions of the intestinal mucosa due to the degradation of polysaccharides by bacteria [11]. In addition, the microbiota has been implicated in the immune response as demonstrated in mouse experiments [12].

The gut microbiome possesses effects on microbiological resistance, for example in regulating the physiology of metazoans, multicellular eukaryotic species considered within the group of microorganisms [13]. This implies that a better understanding of the microbiota could avoid the use of synthetic agents such as antibiotics in the fight against pathogens. This coincides with the vision of consumers to produce healthier animals and decrease the promotion of resistance genes [14]. On the other hand, the interactions between dominant microorganisms and variations within the microbiota have been scarcely described in animals, where the multiple variants, including genetic and functional, and diverse life spans between individuals make it even more difficult to assess. Therefore, more studies are required to compile and structure the information generated and thus help to improve animal production and its derivatives in an economic and healthy environment that ensures a benefit to the animal [15]. The present review aims to describe the main effects of experimental diets in production animals, according to changes in the composition of the basal diet and inclusion of feed additives, and their relationship with the proliferation or inhibition of different microorganisms present in the intestinal microbiome. A summary of the diets can be found in the supplementary material (Table S1).

## 2. Microbiome

The microbiome is the interaction between the different microbial groups and the surrounding internal environment. It is made up of the microbiota, which includes microorganisms such as bacteria, archaea, protozoa, fungi, cyanobacteria, and algae [16]. These interact in a homeostasis within the organism in a complex system where, by any alteration, microbial populations can trigger chains of metabolic reactions that influence at a structural level and affect in a physiological way the development and growth of intestinal mucosa [17]. These components interact within the organism in a multi-complex system, maintaining a state of homeostasis. However, by any alteration, microbial populations can trigger cascades of metabolic reactions that profoundly influence the structural and physiological aspects, ultimately affecting the development and growth of the intestinal mucosa [18]. The composition of microbial populations is diverse; for example, it is estimated that just within the human gastrointestinal tract live about 100 trillion microorganisms, which are taxonomically classified by genus, family, order, and phylum [19]. The onset of microbiome formation is not entirely clear, theorizing that newborn mammals have a sterile microbiome and that it is created at the first interaction outside the womb [20]. It has also been indicated that in mammals colonization begins at birth and starts with the colonization of aerobic and facultative anaerobic bacteria in the first instance through the passage of the vaginal canal, which can vary between species and leaves doubts in cases where cesarean sections are performed [21]. In turn, it is established that initial colonization is fundamental for the development of the neonate; for example, in ruminants the period from birth to weaning is of great importance for the colonization of the microbiome in the rumen and is linked to the concept of coevolution between the microorganisms and the individual [22]. It has also been pointed out in other studies, based on the immune metabolism and microorganisms, that stress caused at weaning can influence immune development and cause a dysbiosis in the microbiota that alters the homeostasis between microorganisms with the promotion of pathogenic colonies [23]. Other studies add concepts such as the “social microbiome”, highlighting that individuals of the same group have a greater similarity between their microbial populations compared to those that do not share the same environment and, therefore, lacking interactions. However, these studies also indicate that diets, genetics, and geographical locations contribute to similarities in microbial composition between species, suggesting that it is not necessarily related to the social transmission of microorganisms between individuals [24]. Additionally, it has been established that factors associated with captivity, such as zoos, farms, and laboratories, can trigger metabolic problems, and modify digestion and reproduction by increasing stress [25]. So, comparing the same species that have been placed in captivity or in the wild, differences in the relative abundance of bacterial taxa can be found [26]. However, it has been determined that the main influencing factor on the microbiota is the diet used in animals, whether in captivity or in a controlled environment [25]. And to a lesser degree it has been reported that contact with soil, water or other exogenous elements contribute to the conformation of the microbial community; for example, in ruminants it has been described that up to 3% of the microbial community is composed of these exogenous microorganisms [27].

Studies that have focused on feed diets that influence the microbiome of production animals are diverse, encompassing those that use modifications of the standard diet. For instance, in the case of ruminants, different proportions of forage and concentrate, as well as the substitution of certain typical constituents, exemplify the diverse dietary adjustment made [28]. On the other hand, there is research that analyzes the change of the microbiome using specific supplements in diets, such as additives, hormones, amino acids, probiotics and vitamins. To help exemplify productive animals, the following is a review of experimental diets that influenced the microbiome ordered according to type and species. The studies are categorized into two groups: firstly, those that made modifications to the basal diet, involving alterations in composition ratios, the addition of foods with distinct qualities, or replacement of specific components. Secondly, we explore those investigations that added supplements, such as probiotics, hormones, amino acids or vitamins, among others relevant factors.

## 3. Ruminant Animals

Ruminants possess three pre-stomachs: reticulum, rumen, and omasum, and a true stomach called the abomasum [29]. The function of the different compartments is diverse and highly specialized. The feed once ingested by the ruminant enters the esophagus and then the pre-stomach (rumen, reticulum, and omasum) where a process of fermentation and decomposition occurs, and then enters the abomasum where chemical and enzymatic digestion occurs [30]. The rumen is constituted by different microorganisms and is where volatile fatty acids are generated in addition to other proteins for the ruminant in the fermentation process carried out especially by archaea. On the other hand, it is important to highlight that the whole process involves the use and expulsion of nitrogen into the environment in the form of methane gas (CH_4_), which has no nutritional value for the animal and so it is eliminated as a by-product of rumen fermentation, a product of the archaea using carbon dioxide (CO_2_) and hydrogen (H_2_) present in the rumen after fiber degradation [31]. At least 65% of emissions of pollutant gases such as methane gas that contaminate the terrestrial atmosphere are produced by the livestock sector, most of them produced by ruminants, and cattle destined for meat or milk production being mainly responsible as generating most of the CH_4_ emissions [32]. Studies have highlighted that there are communities of microorganisms responsible for lower methane gas elimination in ruminants; specifically in studies with lambs it was described that *Fibrobacter* spp., *Kandleria vitulina*, *Olsenella* spp., *Prevotella bryantii*, *succinate*,and *Sharpea azabuensis* are associated with lower H_2_ production [33]. In turn, studies in dairy cows show that CH_4_ emission is mainly linked to H_2_-producing bacteria such as *Ruminococcaceae*, *Christensenellaceae*, and *Lachnospiraceae*, and that in the high CH_4_ emission group, *Succinivibrionaceae* and *Methanosphaera* spp. communities are found in lower abundance [34]. Thus, research to reduce CH_4_ emissions is a latent problem, for which different alternatives have been evaluated [35].

In comparison with monogastric and polygastric animals, ruminants have a great advantage; they are efficient in converting cellulosic matter into energy, as they have a more specialized fermentative digestion. As mentioned above, digestion is developed in regions lacking oxygen, providing an anaerobic environment necessary for the proliferation of microorganisms. In addition, facultative aerobic microorganisms interact to help reduce the oxygen coming from the outside [36]. The temperature inside the rumen ranges between 38 to 42 °C and the pH is normally between 5.5 to 7.0, depending on the diet. The microorganisms present are composed of bacteria, archaea, protozoa and fungi, of which bacteria have a concentration between 10^10^ to 10^11^ bacteria per milliliter in the rumen [37].

Archaea are a group of microorganisms similar to bacteria discovered in 1977 by Carl Woese; since then, more varieties have been discovered and have been grouped to date into 20 phyla in total [38]. Among those classified as methanogens, that is, CH_4_ producers, the genera *Methanobrevibacter gottschalkii* and *Methanobrevibacter ruminantium* can be highlighted, which comprise 74% of rumen archaea [39]. The rumen archaea generally use H_2_ for the production of CH_4_; it is estimated that a reduction or inhibition of the archaea in the rumen would produce an increase in H_2_ that would affect the functions of the enzymes, reducing the efficiency in the conversion of food into nutrients [40]. However, it is known that there are varieties that can use formate, acetate, methyl compounds, and ethanol as an alternative to H_2_ [39].

The protozoa present in the rumen are in a concentration between 10^4^ and 10^6^ per milliliter, reaching half of the microbial mass, and are responsible for 30 to 40% of the total fiber digestion. They are classified in two groups: entodinomorphic and holotrophic, fulfilling mainly a fibrolytic role. Recent research has described that they can produce pectin esterase, cathepsin and glycosyl hydrolase enzymes, also playing a predatory role for bacteria and fungi [41]. The concentration of fungi in the rumen is 10^3^ and 10^6^ zoospores per milliliter, belonging to the class *Neocallimastigomycetes*, being anaerobic fungi found in the digestive tract composed of six genera: *Anaeromyces*, *Caecomyces*, *Cyllamyces*, *Neocallimastix*, *Orpinomyces*, and *Piromyces*. They possess amylolytic and proteolytic activity, their main function being fiber digestion by degrading structural polymers of plant origin [37,42].

### 3.1. Cattle

**Figure 1:**
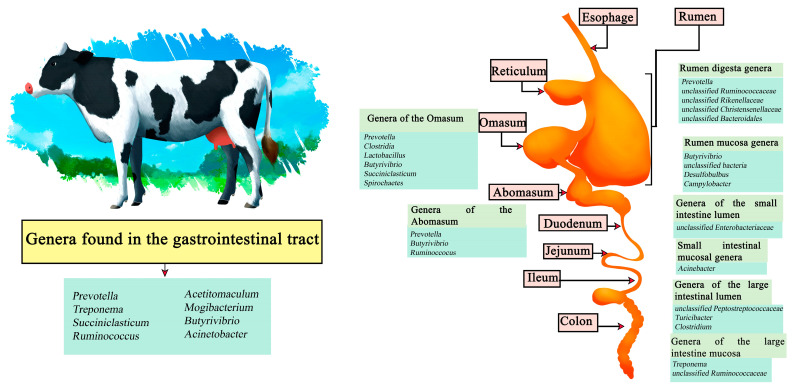
Digestive system of cows. Microorganisms frequently found in different sections of the gastrointestinal tract compartments.

Cattle are one of the main sources of food worldwide, both in the meat and dairy businesses, with meat being a source of protein whose demand is increasing [43]. By 2021, beef production reached a total of 58.152 million tons, where the United States is the largest beef producer reaching 12.730 million tons followed by Brazil and China [44]. By 2030, beef production is expected to reach 44 million tons worldwide [45]. On the other hand, in 2021, dairy production reached a production of 544.072 million tons of cow’s milk, with the European Union being the main producer with 145.700 million tons, followed by the United States and India [46]. Demand for dairy products is estimated to increase over the next 50 years [47]. In cattle it has been described that *Prevotella*, *Treponema*, *Succiniclasticum*, *Ruminococcus*, *Acetitomaculum*, *Mogibacterium*, *Butyrivibrio*, and *Acinetobacter* are common genera that can be found throughout the gastrointestinal tract. In turn, genera such as *Prevotella*, *unclassified Ruminococcaceae*, *unclassified Rikenellaceae*, *unclassified Christensenellaceae*, and *unclassified Bacteroidales* have been reported in the rumen digesta, and *Butyrivibrio*, *unclassified bacteria*, *Desulfobulbus*, and *Campylobacter* in the rumen mucosa. In the Omasum the genera *Prevotella*, *Clostridia*, *Lactobacillus*, *Butyrivibrio*, *Succiniclasticum*, and *Spirochaetes* have been found, and in the abomasum, *Prevotella*, *Butyrivibrio*, and *Ruminoccocus* have been described. Other genera such as *unclassified Enterobacteriaceae* have been described in the small intestine lumen and *Acinebacter* in samples of the small intestine mucosa. Finally, in the large intestine section, *unclassified Peptostreptococcaceae*, *Turicibacter*, *and Clostridium* genera have been seen in lumen samples and *Treponema* and *unclassified Ruminococcaceae* in the mucosa (Figure 1) [48,49].

Concentrations of forage and concentrate can alter the microbiota of cattle. An investigation developed by Wang et al. (2020) in Holstein cows used two diets, one containing a high proportion of forage (70%, high forage, HF) and another with 30% concentrate (high concentrate, HC), on a dry matter basis. There it was shown that a diet rich in forage (HF) has an effect on the diversity of microorganisms; these changes were due to the low pH present at the rumen level, which affects some bacteria. The results for both treatments indicated that the phylum with the highest relative content was *Bacteroidetes*, followed by *Firmicutes* and *Proteobacteria*, with no changes between treatments. On the other hand, the *Prevotella* genus was more abundant in diets rich in fiber (HF), which have the capacity to degrade hemicelluloses, starch, and proteins. Furthermore, in HF diets, the family *Ruminococcoccaceae*, *Veillonellaceae*, and the genus *Selenomonas* were found to be more abundant, suggesting that their abundance is due to a highly fermentable diet [50].

Regarding the use of seaweeds in experimental diets of dairy cattle, *Ascophyllum nodosum* was incorporated as a dietary iodine fortifier in the diet of Holstein Friesian cows. Iodine is a trace element constituent of triiodothyronine and thyroxine hormones, and its shortage can lead to health problems in populations. The implementation of the diet was carried out in cows using a total mixed concentration (TMR) based on: corn silage (58%), sainfoin hay (13%), second-cut alfalfa hay (6%), and concentrate (23%). One treatment considered this concentrate as a control (CC) and another experimental treatment included *Ascophyllum nodosum* concentrate (EC). The results showed that the inclusion of *Ascophyllum nodosum* presented the microbial populations at phylum level in the following proportions: *Firmicutes* (57.14%), and *Proteobacteria*, *Bacteroidetes* and *Actinobacteria* at 14.28% each. Seven Operational Taxonomic Units (OTU) were identified in the milk. At the genus level, *Lactococcus*, *Pseudomonas*, and in smaller quantities *Staphylococcus*, *Enterococcus*, *Clostridium*, *Bacteroides*, *Actinobacteria*, and *Microbacterium* were found, with a smaller quantity of *Pseudomonas*, *Lactococcus raffinolactis*, *Staphylococcus* spp. and *Pseudomonas jessenii*. However, a higher population of *Lactococcus lactis* and *Lactococcus garvieae* was found [51].

Several studies have been conducted with experimental diets in Holstein dairy cows. One of them used the yeast *Saccharomyces cerevisiae*, a type of fungus commonly used in industrial processes and that provides health advantages in adequate doses [52]. This yeast was used to modify ruminal fermentation and maximize forage yield. The observed results showed an increase in populations of cellulolytic bacteria highlighting *Ruminococcus flavefaciens* and *Fibrobacter succinogenes* that possess specific mechanisms for cellulose adhesion and breakdown [53], accompanied by a reduction in lactate-producing bacteria such as *Streptococcus bovis*. This shift among populations led to an increase in volatile fatty acids (VFA) and an increase in the amount of protein due to a decrease in the protozoan *Entodinium*, which is responsible for ingesting bacteria and reducing the microbial protein supply to the small intestine. Therefore, the use of *Saccharomyces cerevisiae* provided higher energy efficiency and better nitrogen utilization [54].

A study by Kasparovska et al. (2016) proposed using isoflavone extract (12.5 g) as a dietary supplement in lactating cows of the Fleckvieh x Holstein cross, a type of phytoestrogen found in legumes and reported to support milk production in ruminants [55]. The results of this experiment indicated that, in the supplemented experimental group, the proportion of *Bacteroidetes* was higher than that of *Firmicutes.* At the family level the populations found in the experimental groups were *Prevotella*, *Fibrobacteraceae* and *Burkholderiales*. However, in control animals it was found that in populations of the phylum *Firmicutes*, the orders *Clostridiales*, *Erysipelotrichales* and *Lactobacillales* were more abundant than in experimental cows [56].

Yoshimura et al. (2018) posited that the use of three components in an experimental diet: flaxseed oil, propolis, and vitamin E, can have a synergistic effect in Holstein dairy cows. Flaxseed oil is rich in α-linolenic acid (18:3), a fatty acid known for its beneficial effects [57]. The experiment was conducted on three groups of supplemented cows; a control diet (25 g flaxseed oil/kg DM (FO); a diet with 25 g flaxseed oil/kg DM and 1.2 g propolis-based product/kg DM (PBP); and a diet with flaxseed oil, PBP and 375 IU vitamin E/kg DM (PBP-E). The results indicated that the diet supplied with flaxseed oil (FO) produced a decrease in protozoa, with a greater number of *Entodinium* and a lower number of *Isotricha*, an increase in bacterial species *Butyrivibrio fibrisolvens*, and a reduction in the populations of *Anaerovibrio lipolytica* and *Methanobrevibacter ruminantium*. Therefore, PBP and PBP-E treatments did not have a significant impact on microbial populations. However, they possess positive effects on milk quality by antioxidant activity and do not affect digestive parameters [58].

### 3.2. Sheep

**Figure 2:**
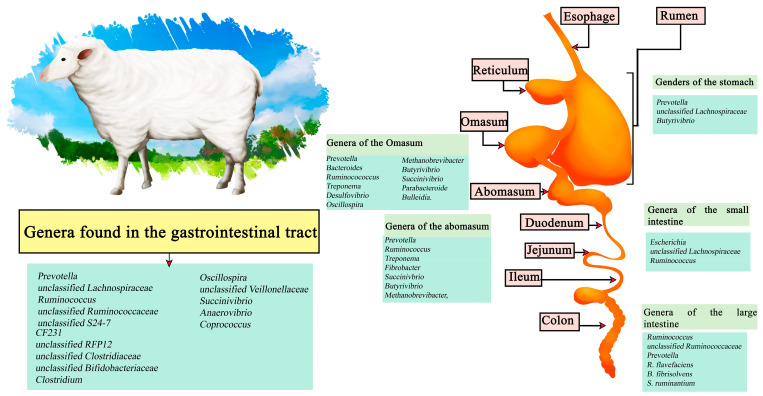
Digestive tract of sheep. Microorganisms frequently found in different sections of the gastrointestinal tract compartments.

The sheep meat market includes the sale of meat, milk, wool, and skin, depending on the country of origin. Worldwide, there are an estimated 1 billion sheep, distributed mainly in northern Europe, Asia, South America, Australia, and New Zealand, with a consumption of 2.5 kg per person per year. In general, sheep production systems are classified into 3 categories: extensive production, intensive production, and traditional grazing; the latter has been reduced mainly due to the advance of agriculture that has reduced the available space, in addition to problems in yield [59]. In sheep studies the predominant microorganisms in the regions encompassing the sheep stomach have been *Prevotella*, *unclassified Lachnospiraceae*, *and Butyrivibrio*. In the abomasum these are *Prevotella*, *Ruminococcus*, *Treponema*, *Fibrobacter*, *Succinivbrio*, *Butyrivibrio*, and *Methanobrevibacter*, while in the Omasum *Prevotella*, *Bacteroides*, *Ruminocococcus*, *Treponema*, *Desulfovibrio*, *Oscillospira*, *Methanobrevibacter*, *Butyrivibrio*, *Succinivibrio*, *Parabacteroide*, and *Bulleidia* have been described. On the other hand, the three most dominant genera in the small intestine are *Escherichia*, *unclassified Lachnospiraceae*, and *Ruminocococcus*; in the small intestine *Ruminocococcus*, *unclassified Ruminococcaceae* and *Prevotella*. *R*. *flavefaciens*, *B*. *fibrisolvens* and *S*. *ruminantium* are found. Predominant throughout the gastrointestinal tract are the genera of *Prevotella*, *unclassified Lachnospiraceae*, *Ruminococcus*, *unclassified Ruminococcaceae*, *unclassified S24-7*, *CF231*, *unclassified RFP12*, *unclassified Clostridiaceae*, *unclassified Bifidobacteriaceae*, *Clostridium*, *Oscillospira*, *unclassified Veillonellaceae*, *Succinivibrio*, *Anaerovibrio*, and *Coprococcus* (Figure 2) [60,61].

Alfalfa (*Medicago sativa*) is an excellent forage. However, its availability is limited. A study sought to replace alfalfa with native grass in the diet of Ujimqin lambs. For this purpose, three experimental diets were used: a high percentage alfalfa diet (30%, HA) with 10% native grass; a moderate percentage alfalfa diet (20%, MA) with 20% native grass; and a low percentage alfalfa diet (10%, LA) with 30% native grass. It was found that the proportions of microorganisms that constitute the microbiota in the three treatments were different, showing no significant differences. However, the presence of the *Prevotella*, *Muribaculaceae*, and *Rikenellaceae* gut groups, and abundance of the *Bacteroidales* gut group in MA diets was highlighted [62].

Forage oilseed rape (*Brassica napus*) is the second most important oilseed crop in the world used for research in breeding studies. It is used in crop rotation in Europe, Australia, Canada, and China, in turn helping to maintain soil fertility achieving sustainable production [63]. An experiment designed by E. Du et al. (2022) used castrated male lambs of the Hu breed and supplemented them with diets based on total mixed ration (TMR) with different inclusion levels of forage canola, from 0, 100, 200, 200, 300 and 400 g/kg^−1^, in order to know the effects on meat quality and the changes caused in the microbiome. Results at the phylum level indicated that *Bacteroidetes* and *Firmicutes* accounted for ~92.5% of the total taxa and dominated all rumen microbial communities. In contrast, *Proteobacteria*, *Patescibacteria*, *Spirochaetes*, *Tenericutes*, and *Euryarchaeota* were less abundant, representing 0.1–10.0% of the total bacteria for all lambs. In addition, among the cellulose degrading microorganisms, the following genera were found *Fibrobacter*, *Eubacterium* and *Ruminiclostridium*. The conclusion was that the inclusion of forage canola increases the levels of amino acids and intramuscular α-linolenic acid in terms of meat quality, in addition to promoting the growth of cellulolytic bacteria in the rumen [57].

Antioxidants can help to combat the stress caused by dietary changes in young animals. Grape pomace (*Vitis vinifera* L. *var. Moschato*) has been proven as an important source of polyphenols that can act as powerful antioxidants in lambs [64]. Research by Kafantaris et al. (2017) used grape pomace in male Chios lambs to use polyphenols as prebiotic supplements to improve intestinal health. For this purpose, they used diets containing a standard diet for the control group. The experimental group used a diet containing silage with grape pomace polyphenolic additives, based on: corn silage (51% solids), grape pomace (9%), and water (40%). The results indicated a reduction in *Enterobacteriaceae* in the fecal microflora, accompanied by a reduction in *Escherichia coli*, as well as other pathogenic bacteria. In turn, the population of *Bifidobacterium*, beneficial bacteria in polyphenol treatment, increased [65].

Energy and protein levels in diets are capable of altering rumen growth and development by modifying its physiological and anatomical properties, which affects milk quality and production. When high quality forage such as corn silage and alfalfa hay are ingested, the productive performance of ruminants can be increased [66]. One study used Hu Chinese lambs randomized in a 2 × 2 factorial arrangement with a first factor of high metabolizing energy (HE) and low metabolizing energy (LE), with 4 treatments of high protein (HP) and low protein (LP) for the second factor, respectively. The results detected 16 phyla, highlighting *Bacteroidetes* as the most dominant, followed by *Firmicutes*, Proteobacteria and Spirochaetes. At the genus level, 126 were identified, highlighting *Prevotella*, the gut group, *Succinivibrionaceae*, and *Ruminococcaceae*, present in all treatments. At the genus level, the dominant members of the high metabolizing energy (HE) group samples contained mainly *Veillonellaceae*, *Succinivibrionaceae*, and *Veillonellaceae*. Low metabolizing energy (LE) groups were dominated by *Bacteroidales* and *Lachnospiraceae*. The results obtained give a profile of bacteria present in the microbiome under varying energy and protein, thereby offering a valuable profile for future studies [67].

In order to improve gut health in an economical manner, the effects of rumen-protected methionine (Met) and lysine (Lys) were studied. These were used in experimental low protein (LP) diets, which have been shown to be able to reduce costs associated with protein sources and reduced nitrogen emissions in varied studies, such as chickens and pigs [68,69]. Gebeyew et al. (2021) used male Hulunbuir lambs fed diets with normal protein (NP), low protein (LP) and an LP diet supplemented with Met and Lys (LP + RML). The results indicated that LP + RML diets increased the *Paenarthrobacter* population in the jejunum and ileum. *Ruminococcus* and *Clostridium* were reduced in the jejunum and enriched in the ileum; *Saccharimonas* decreased in the duodenum, jejunum and ileum; and *Gastranaerophilales* proliferation increased in the duodenum. This study concluded that the inclusion of methionine and lysine does not result in adverse alterations in the microbiota gut, conversely, contributes to a healthier microbial community [70].

### 3.3. Goats

**Figure 3:**
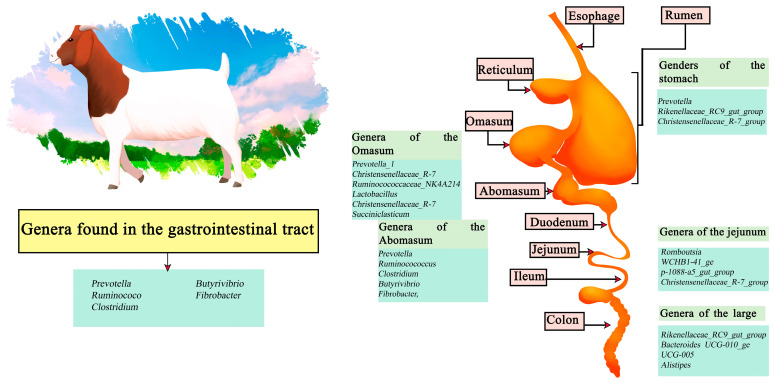
Digestive tract of goats. Microorganisms frequently found in different sections of the gastrointestinal tract compartments.

Goats are small ruminants that have been domesticated since ancient times; their uptake is more popular in the East and reduced in the West. However, they have been gaining more relevance recently [71]. The cause of their popularity is due to the healthier nutritional qualities present in goat meat (Chevon), containing a lower level of fat, saturated fat and cholesterol. It also has a higher content of polyunsaturated fatty acids (PUFA), being healthier than other red meats [72]. Among the genera identified in the stomach are *Prevotella*, *Rikenellaceae_RC9_gut_group*, and *Christensenellaceae_R-7_group*. Meanwhile, the abomasum is composed of *Prevotella*, *ruminocococcus*, *Clostridium*, *butyrivibrio*, and *fibrobacter*, followed by, in the omasum, *Prevotella_1*, *Christensenellaceae_R-7*, *Ruminocococcaceae_NK4A214*, *Lactobacillus*, *Christensenellaceae_R-7*, *Succiniclasticum*, *Clostridiales_vadinBB60*, and *Clostridiales_vadinBB60*. The jejunum section is dominated by *Romboutsia*, *WCHB1-41_ge*, *p-1088-a5_gut_group*, and *Christensenellaceae_R-7_group*. Finally, the large intestine region presents a higher abundance of the genera *Rikenellaceae_RC9_gut_group*, *Bacteroides UCG-010_ge*, *UCG-005*, and *Alistipes* [73]. In contrast, along the intestine the main genera involved are *Prevotella*, *ruminococcus*, *Clostridium*, *Butyrivibrio*, and *Fibrobacter* (Figure 3) [74,75].

Milk from ruminants is an important source of nutrients providing protein, vitamins, and minerals. However, goat milk stands out for its nutritional qualities, being a good source of potassium, calcium, and phosphorus. In addition, it possesses many fatty acids, such as conjugated linoleic acid (CLA), these being a mixture of isomers of linoleic acid and alpha linoleic acid that enhance the health properties of milk [76,77]. A study conducted in alpine goats used hemp seeds (linoleic acid) and flaxseeds (alpha linoleic acid) as feed supplements. Three treatments were carried out: control diet; diet supplemented with flaxseed; and diet supplemented with hempseed. The results for the two experimental treatments showed the predominance of *Bacteroidetes* and *Firmicutes* phyla with a high abundance of *Prevotellaceae* and *Veillonellaceae* and a low abundance of *Ruminococcaceae*, *Prevotellaceae*, and *Lachnospiraceae*. The *Prevotellaceae* family was dominant in the experimental diets at both sampling times. In all treatments, the genus *Methanobrevibacter* was the main Archaea found. At the genus level, flaxseed supplementation increased *Succinivibrio* spp. and *Fibrobacter* spp. populations, while decreasing the relative abundance of *Prevotella* spp. Based on this research, it is manifested that fatty acid intake in the diet of alpine goats modifies the intestinal microbiome, which would also affect the fatty acids presented in milk [78].

The ingestion of medicinal plants has the potential to modify ruminal fermentation. In a study developed by Yusuf et al. (2017) it was proposed to used *Andrographis paniculata* (AP) for this purpose. This medicinal plant is of Asian origin, belongs to the *Acanthaceae* family and is used in humans to treat inflammatory diseases, having antioxidant properties [79]. The research used Boer goats, providing a control diet (basal diet without additives) plus two experimental diets: a basal diet +1.5% (*w*/*w*) of *Andrographis paniculata* leaf powder (APL); and a basal diet +1.5% (*w*/*w*) of *Andrographis paniculata* with whole plant powder (APW). The results found that the treatment that included *Andrographis paniculata* powder favored the growth of the genus *Ruminococcus* (*albus*, *flavefaciens*) and *Fibrobacter succinogenes* due to a higher ruminal pH [80].

In the search for alternatives to the use of antibiotics, Zhou et al. (2020) described as an alternative the use of bioactive compounds or essential oils from plants as a viable option. This approach is considered environmentally safe and cost-effective. The researchers proposes lolot (Piper sarmentosum, PSE) as a medicinal plant, due to its high number of bioactive compounds and antimicrobial activity. The study was conducted on Hainan black goats and employed four treatments with 0, 300, 600 or 1200 mg/kg of PSE extract with zeolite powder. The results showed a decrease in protozoa, fungi, *Ruminococcus flavefaciens*, and *Fibrobacter succinogenes* in the 1200 mg/kg PSE treatment. However, the populations of *Ruminococcus albus*, *methanogens*, and *Butyrivibrio fibrisolvens* present in the rumen were not altered by the treatment. Therefore, the experimental diets provided antioxidants properties to the goats, affecting the microbiota populations [81].

To evaluate the effects produced by rice intake in goats, one study used Liuyang Black goats with a control treatment (concentrate/hay 55:45) and a rice-rich diet (concentrate/hay 90:10, HR) were compared. Rice supplementation resulted in a decrease in ruminal pH, followed by an increase in the populations of the genus *Ruminococcaceae* and a decrease in *Christensenellaceae* and *Bacteroidales*, resulting in a loss of gut microbial diversity [82].

Methionine is an important amino acid for the correct nutritional balance in production animals, mainly in ruminants. Upon reaching the rumen, not all amino acids supplied can be absorbed correctly, so dietary protected proteins are used, which have shown improvements in milk production parameters and growth rates [83]. In addition, there are methionine analogues such as HMB (2-hydroxy-4-(methylthio)-butanoic acid) and isopropyl ester HMBi, which are usually supplied at the post-ruminal stage. HMBi can consistently provide methionine by degrading at slower rate in the rumen. In addition, it provides multiple benefits in terms of biological activity, nutrient digestibility, and milk composition. Chen et al. (2020) tested the effects of HMBi on different parameters in Xiang Dong black goats. They used a control supplied by a basal diet and implemented with HMBi at the following proportions: 0%; 0.05%; 0.10%; and 0.20%. At the microbiota level, the most frequently found phylum was *Bacteroidetes* followed by *Firmicutes*; at the genus level, 86 taxa were identified, including: *Prevotella*; *Succinivibrio*; *Selenomonas*; *Succiniclasticum*; YRC22; and *Ruminococcus*. Correlation analysis determined that *Succinivibrio*, *Bilophila*, and *Ruminobacter* could be related to increased feed intake, although further studies are needed to determine their role. It was concluded that methionine administration had no detrimental effects on goats [84].

## 4. Monogastric Animals

Monogastric animals have a similar gastrointestinal system in many respects, being classified into carnivores, herbivores, and omnivores. However, there are structural differences between them, for example in the cecum and/or colon. Since they do not have a stomach for fermentative digestion, these structures (cecum and colon) have developed in greater proportion. They are larger in herbivores, as they require bacterial fermentation of fiber [85]. In turn, the microorganisms present in the microbiome are dependent on multiple factors including diet. However, external factors like temperature can cause heat stress in animals, where in animals which, in turn, affects the intestinal composition and microbiota. Animals with absence of sweat glands such as chickens and pigs, experience differences in their bacterial composition as a result [86]. Other sources of stress such as confinement, geographical location, husbandry systems or factors such as breed and age are also determinants [87]. Therefore, the microorganisms found among monogastrics are different from each other.

### 4.1. Chickens

Within poultry, chicken meat (*Gallus gallus domesticus*) provided from broilers is one of the most consumed food products in the world, with a high demand due to its nutritional properties with a high content of protein, B-complex vitamins and minerals, and a low level of saturated fats. Its demand is expected to be increasing with the growing increase in population [88].

**Figure 4:**
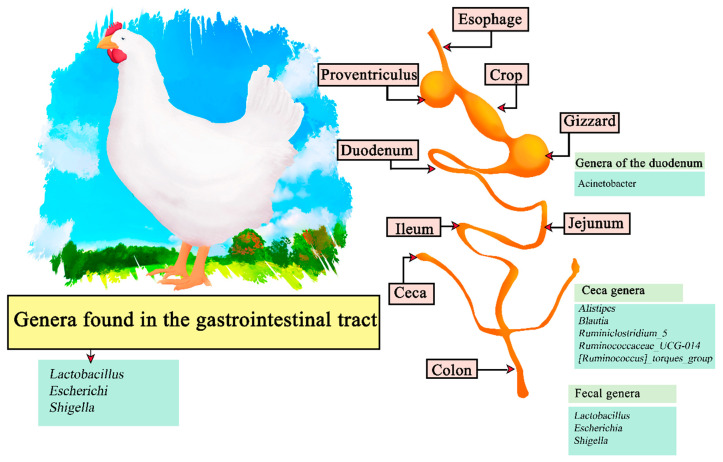
Digestive tract of chickens. Microorganisms frequently found in different sections of the gastrointestinal tract compartments.

The digestive system of poultry consists of the oral cavity (beak), esophagus, crop, stomach (constituted of two parts; proventriculus and gizzard), small intestine (composed of duodenum, jejunum and ileum), liver, cecum, large intestine, and cloaca (Figure 4). The ingested food is hydrated, ground into small particles, acidified and attacked by endogenous enzymes to obtain the macronutrients. The stomach has oxyntopeptic cells responsible for secreting both hydrochloric acid and pepsinogen, and lipase resulting from reflux from the duodenum can also be found. The functions of the proventriculus and gizzard (ventriculus) are, respectively, to initiate primary digestion using digestive enzymes and to exert a mechanical function by grinding the food to a suitable size to continue through the digestive tract [89,90]. The pH values present in some areas of the digestive system are: beak (6.7); crop (6.4); ileum (6.7); rectum (7.1). As described, the pH in chickens is more acidic than in other mammals and does not change during the life span. On the other hand, the constituent microorganisms of the microflora are obtained by the interaction of newly hatched chicks and the eggshell surface, changing over time [91]. It has been recorded that *Clostridium_sensu_stricto_1* is predominant in newly hatched chicks in the cecum, duodenum, and feces. However, *Lactobacillus* is more abundant in later stages such as after 35 days. Therefore, the genera found in feces are *lactobacillus* and *Escherichia-Shigella*, in the cecum *Alistipes Blautia, Ruminiclostridium_5, Ruminococcaceae_UCG-014*, and the *[Ruminococcus]_torques_group*, and finally the genus *Acinetobacter* in the duodenum [92]. Among the most recurrent genera within the gastrointestinal tract are *Lactobacillus, Escherichi,* and *Shigella* (Figure 4) [93].

The perennial plant *Achyranthes japonica*, known in oriental countries as a medicinal plant, stands out for its anti-inflammatory, hepatoprotective, antioxidant and anticarcinogenic functions. Its active ingredients such as saponin, phytoecdysteroids, 20-hydroxyecdysone, triterpenoids and inokosterone have been used in animals such as pigs [94]. Recent research proposed the use of the active components present in Achyranthes japonica (AJE) as a feed supplement in Ross 308 broiler chickens [66]. Therefore, four experimental diets were used, a basal diet (CON), followed by treatments containing both the basal diet and different percentages of AJE extract: T1 (0.025% AJE), T2 (0.05% AJE), and T3 (0.1% AJE). In the fecal microflora, it was found that the *Lactobacillus* increased as AJE increased, while *E*. *coli* and *Salmonella* populations decreased as AJE increased. Thus, Park & Kim (2020) conclude that diets with AJE can improve parameters in broilers, such as growth performance, dry matter digestibility, and nitrogen.

A study by Hafsa et al. (2018) used grape seeds, rich in polyphenols, as a dietary additive in Cobb-500 broiler chickens. Subsequently, their ileum was removed and digestive contents were collected at 42 days of age. Results indicated a reduction in *E*. *coli* and *Streptococcus* populations in the ileum and an increase in ileal populations of beneficial bacteria such as *Lactobacillus*. It was reported that polyphenols may have bacteriostatic or bactericidal action, or may act to inhibit the adhesion of infection-causing bacteria within the cells of the intestinal tract. Thus, the active components of herbal derivatives, including grape seeds, could help prevent the growth of pathogenic bacteria and promote the population of non-pathogenic bacteria, like *Lactobacillus* spp. and *Bifidobacterium* spp. In the particular case of *Lactobacillus*, possess the ability to metabolize phenolic compounds supplying energy to the cells and positively impacting bacterial metabolism [95].

An alternative to replace food of plant origin consists of using flour from *Tenebrio molitor* larvae (TM flour). This worm, also known as yellow mealworm, has been one of the most used insect species in feed due to its high nutritional value, with a high protein and lipid content. It is also used in production animals. In a study conducted by Rumbos et al. (2020), Arbor Acres broilers were used and subjected to various diets during the rearing phase. The first of these was corresponding to the initial phase (first 10 days), a second corresponding to the growth phase (11 to 25 days), followed by a finishing diet (26 to 42 days). The study included a control and two experimental diets that replaced 2.5% (MT 2.5) and 5% of the basal diet with TM meal (MT 5). The results showed that the feed conversion rate was higher in the control than in the 2.5% MT treatment. However, no changes were observed in the other treatments in the rearing phases. On the other hand, samples obtained at day 25 from the cecal region indicated a reduction in *E*. *coli* content in the 5% TM treatment, demonstrating a linearity of *E*. *coli* reduction at higher TM supplementation. It is concluded that TM intake improves immune activity due to a prebiotic effect found in chitin from larvae [96].

Xylanase is a carbohydrase used to improve feed digestibility and has been used in broilers to facilitate digestion of dietary fiber [97]. An investigation tested the effect of xylanase inclusion in ROSS 308 broilers by applying four experimental diets: Control (CON), control plus xylanase (XYL); control plus emulsifier (EMU); and control plus xylanase and emulsifier (XYL + EMU). These diets were administered according to an initial phase (1 to 11 days), emphasizing the use of corn and rapeseed oil as supplementary fat, and in the growth and finishing phases (from 11 to 25 and from 25 to 42 days) beef tallow was integrated. Analyses showed that there were no significant differences in the populations of *Bifidobacterium*, *Lactobacillus*, and *Escherichia coli* in the treatments containing xylanase or emulsifiers. On the other hand, *Clostridium* (butyric acid-producing bacteria) showed a decreased relative abundance in the emulsifier-containing groups. However, the other butyric acid-producing bacteria were largely unaffected by emulsifier treatment. Consequently, fermentation in the cecum and enzymatic digestion in the duodenum and ileum were favored upon EMU and XYL ingestion [98].

Currently, there has been increased awareness of the use of growth-promoting antibiotics and their effect on the emergence of resistant strains, so a replacement to these in productive use is being sought. Sophorolipids (SPL) are biosurfactants or biological detergents composed of a hydroxylated fatty acid and a glucose disaccharide (Sophorose) from non-pathogenic yeasts. Due to their biodegradability and low toxicity, the food industry has been focused on their use. [99]. It is proposed that the application in Ross 308 chickens can improve intestinal health and improve the growth performance. A study conducted by Kwak et al. (2021) used three experimental diets in this type of chickens: CON (basal diet); BAM a growth-promoting antibiotic (10 mg/kg bambermycin); and SPL (10 mg/kg SPL). Microbial populations in the SPL diet at the phylum level were enhanced in the *Firmicutes* population over *Bacteroidetes*. At the genus level *Lactobacillus* increased in abundance and *Streptococcus* decreased. At the species level *Lactobacillus helveticus*, *Lactobacillus salivarius* and *Akkermansia muciniphila* showed an increase while *Streptococcus gallolyticus* was reduced in both the BAM and SPL treatments. It was concluded that the SPL diet contributed to improve intestinal defenses and alleviate inflammation, which promotes growth in animals [100].

### 4.2. Pigs

**Figure 5:**
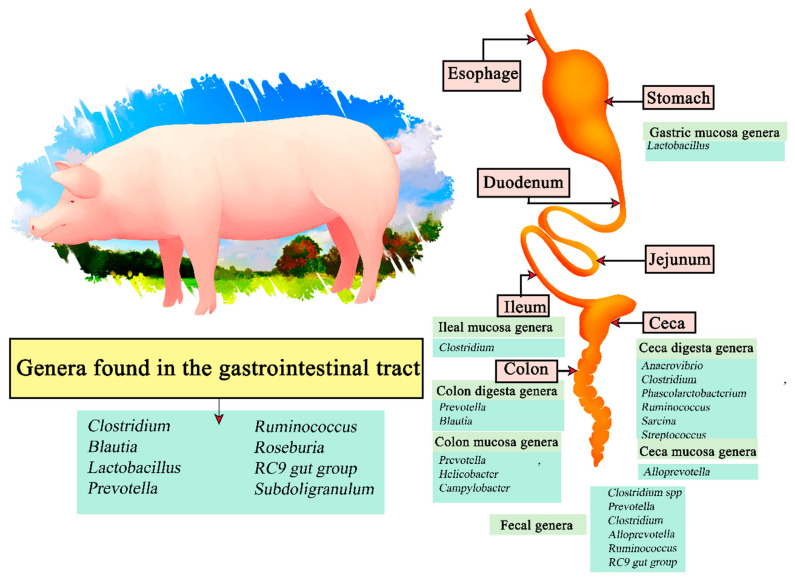
Digestive tract of pigs. Microorganisms frequently found in different sections of the gastrointestinal tract compartments.

Pigs are omnivorous animals and form part of the most consumed type of meat worldwide. Within global meat production, world pork consumption is expected to increase to 129 Mt in ten years, with an estimated 35.6 kg/year of pork consumption by 2030 [101]. Its digestive system is composed of the mouth where the food is introduced, and the stomach which is made up of four regions and includes the esophageal, cardiac, fundic, and pyloric regions (Figure 5). The function of the cardia region is to mix the food by means of a mucus, then the gastric glands of the fundus digest the food with hydrochloric acid and digestive enzymes, and thus the food is directed to the fundus. The pyloric region subsequently secretes mucus into the digestive membranes that serve as protection. Other structures present are the small intestine (duodenum, jejunum, and ileum), where food absorption takes place; the liver secretes bile fluid and the pancreas secretes enzymes, followed by the large intestine constituted by the cecum which digests the fibrous part, and then the colon and rectum. The gastric system found in pigs performs similar functions as in humans. However, it presents some differences in size and shape; the porcine cecum is relatively larger, the porcine colon is spiral oriented and pigs lack an appendix [90,102]. Therefore, the anatomical, physiological, and immunological similarities between humans and pigs mean that the microorganisms present in pigs have similarities to those found in the human digestive system [103,104]. The microorganisms normally found in pigs can be classified into the following phyla: *Firmicutes*, *Proteobacteria*, and *Bacteroidetes*, highlighting the presence of *Firmicutes* phylum populations in the duodenum and jejunum, and of the *Proteobacteria* phylum in the gastric and ileal compartments [105].

The genus *Lactobacillus* was found to be more abundant in the gastric mucosa, and *Clostridium* in samples from the ileal mucosa. *Anaerovibrio*, *Clostridium*, *Phascolarctobacterium*, *Ruminocococcus*, *Sarcina*, and *Streptococcus* were present in the ceca digesta, and *Alloprevotella* in the cecal mucosa. *Prevotella* and *Blautia* were present in colon digesta samples, and *Prevotella*, *Helicobacter*, and *Campylobacter* in the mucosa. Finally, *Clostridium* spp. *Prevotella*, *Clostridium*, *Alloprevotella*, *Ruminococcus*, *RC9 gut group* and *Treponema* were present in fecal samples [106]. Therefore, the genera *Clostridium*, *Blautia*, *Lactobacillus*, *Prevotella*, *Ruminococcus*, *Roseburia*, *RC9 gut group*, and *Subdoligranulum* are present in most of the gastrointestinal tract of swine.

The search for alternatives in the use of carbohydrates in animal diets has become more important due to the limitations of food sources that have been reduced in the agricultural sector due to environmental changes and the problems associated with the decrease in nutrients in the soil, added to the loss of food that is wasted worldwide [107]. Therefore, the search for sustainable carbohydrate alternatives for animal consumption has intensified, and studies have evaluated the administration of processed carbohydrates not suitable for human consumption that fall under the classification of Former foodstuff products (FFPs) [108], in Large White × Landrace pigs using a standard diet for post-weaning piglets for the control group (CTR) and in the experimental group (FFPs) a standard diet including 30% residues of bakery products and wheat-derived varieties as carbohydrate sources. The results indicated that an inclusion of FFPs decreased microbial diversity and decreased the proportion of the genus lactobacillus, and increased the genera of bacteria belonging to the proteobacteria; however, it did not generate major changes in the microbial community and no problems in growth performance were observed [109].

Grape seeds are an important source of flavonoids, which are known for their beneficial effects on health. Polyphenols oxidized in the presence of aflatoxin B1(AF1) can form a complex that helps prevent AF1 from exerting its toxic action in the gut. An experiment conducted by Grosu et al. (2019a) used TOPIGS-40 hybrid pigs and supplemented them with four diets in different treatments: (1) Standard (Control); (2) Diet contaminated with 320 µg/kg AFB1 (AFB1); (3) Diet supplemented with grape seed meal (GC); and (4) Diet with 320 µg/kg AFB1 and 8% grape seed meal (AFB1 + GC). The results indicated, at the phylum level from highest to lowest abundance, the presence of: *Firmicutes*, *Bacteroidetes*, *Proteobacteria*, *Spirochaetes*, *Tenericutes*, and *Actinobacteria*, with traces of *Euryarchaeota*, *Fibrobacteres*, *Cyanobacteria*, and *Deferribacteres*. Although their proportions were altered, for example in the AFB1 + GC treatment, the proportion of *Bacteroidetes* increased, while that of *Firmicutes* decreased compared to the control diet. At the genus level, the treatment including GC increased the populations of *Megasphera*, *Clostridiales*, *Anaerovibrio*, and *Prevotella*, and decreased the genera of *Lactobacillus*, *Lachnospiraceae*, *Bacteroidales*, and *Campylobacter*. The authors noted that the inclusion of GC may help to mitigate the negative effects caused by AFB1 contamination in pig diets. The mechanisms that alter the microbiota by the action of aflatoxin B1 and grape seed meal are unknown [110].

The excessive use of antibiotics in the livestock industry has aroused the interest of researchers in the search for replacements with non-harmful feed additives. In this regard, short-chain fatty acids such as butyrate, whose active element is butyric acid, have been shown to help improve intestinal health by regulating proteins present in the mucosa [111]. Butyric acid can be found in salt form as sodium butyrate or as a triglyceride called tributyrin, both of which are used in humans and animals as prodrugs [112]. A work by Sun et al. (2020) sought to apply sodium butyrate in the diet of pigs using pigs from Yorkshire-Landrace × Duroc boar crosses. The study considered three treatments: (1) basal diet; (2) basal diet + 40 ppm zinc bacitracin; and (3) basal diet + 0.2% sodium butyrate. The results indicated that at the phylum level, *Firmicutes*, *Bacteroidetes*, *Proteobacteria*, *Verrucomicrobia*, *Actinobacteria*, *Tenericutes*, *Cyanobacteria*, and *Synergistetes* were detected in the cecum, with abundance from highest to lowest, respectively. At the genus level, treatment 3, containing sodium butyrate compared to treatment 1, showed a higher relative abundance of *Anaerovibrio*, *Megasphaera*, and *Prevotella*, and a decrease in *Flavobacterium* populations compared to group 1. On the other hand, the populations of the genus *Dorea*, *Blautia*, *Desulfovibrio*, *Coprococcus*, *Succinivibrio*, and *Ruminococcus* were decreased in the group with sodium butyrate, in contrast to group 2 containing zinc bacitracin. The authors proposed that the addition of sodium butyrate to the diet improves the small intestinal mucosa, promoting epithelial cell growth, and increasing villus length. In addition, it decreases carbohydrates and proteins entering the cecum and consequently decreases the relative abundance of *Firmicutes* and *Proteobacteria* [113]. On the other hand, Wang et al. (2019) proposed the use of tributyrin over sodium salt, pointing out that the latter tends to be absorbed by the upper digestive tract, whereas tributyrin can cross this barrier and be absorbed by the lower digestive tract. In this study, different levels of tributyrin were used in pigs (Duroc × (Landrace × Yorkshire)) and a control diet was used followed by three dietary treatments with 250, 500 and 750 mg/kg of tributyrin. The results showed that the use of tributyrin increased the *Lactobacillus*/*E*. *coli* ratio, which is indicative of good intestinal health, thus reducing the negative health effects of *E*. *coli* infection [114].

Apart from the aforementioned methods to replace antibiotics, it is possible to use probiotics such as *Bacillus coagulans* (BC). The latter produces enzymes and organic acids such as lactic and acetic acid [115], which acidify the pH of the intestinal tract, controlling pathogens and yeast hydrolysates that are sensitive to pH. In addition, *Bacillus coagulans* bacteria contain active substances such as β-glucan and mannan which are constituents of the cell wall [116]. Therefore, it is expected that the use of *Bacillus coagulans* can help improve the health of the gastric system in animals and strengthen the immune system by functioning as a prebiotic. In a study by Shin et al. (2019), [Landrace × Yorkshire] × Duroc pigs and three treatments were used: (1) Control diet (CON) and enramycin (20 mg/kg); (2) Diet with *Bacillus coagulans* (20 mg/kg, BC group); and (3) Diet with yeast hydrolysates (3000 mg/kg, YH group). The diet belonging to the BC group significantly increased the number of *Bifidobacterium* in the colon, followed by a decrease in *E*. *coli* counts. The YH diet increased *Lactobacillus* and *Bacillus* counts in the cecum, apart from increasing *Bifidobacterium* and *Bacillus* in the colon. Therefore, an increase in microbial populations beneficial to the host could be detected, demonstrating the immunological role of the probiotic *Bacillus coagulans* [117]. Another probiotic alternative is the use of *Lactiplantibacillus plantarum* proposed by authors Shin et al. (2019), which can enhance and stabilize the microbiota as well as other lactic acid bacteria [118]. The study used pigs (Landrace × Yorkshire × Duroc) with control diets and diets supplied with JDFM LP11 probiotics (2.5 × 10 7 CFU/mL) in a weaning diet. Aliquots of 50 mL/kg diet, 1.25 × 10^9^ CFU/kg diet were fed to the experimental group. The most abundant phyla were *Bacteroidetes* and *Firmicutes* in all diets; at the family level 19 different families of bacteria were found with significant differences in *Prevotellaceae*, *Erysipelotrichaceae*, *Sphaerochaetaceae*, *Spirochaetaceae*, and *Christensenellaceae*, with a higher abundance in *Prevotellaceae* and *Ruminococcaceae*. Therefore, a higher abundance of *Spirochaetaceae*, *Eryspelotrichaceae*, *Sphaerochaetaceae*, and *Christensenellaceae* was found in the experimental diet and a lower abundance of *Prevotellaceae*. It was concluded that the microorganisms involved have effects at the immunological level and on the weight gain of the animals. On the other hand, the experimental diet helped in the development and integrity of the intestinal epithelium [119].

## 5. Fish

Aquatic resources such as fish possess excellent nutritional value as a source of protein, fatty acids, vitamins, minerals, and essential micronutrients [120,121]. In 2020, global fish production from aquaculture reached an all-time high of 214 million tons, distributed among other marine products such as aquatic animals and algae [122]. Efforts in this industry are focused on increasing yields and decreasing costs associated with production by optimizing fish farming. Understanding the microbiome is also fundamental in fish, as it is involved in the development and efficiency of the gastrointestinal tract, resisting infections and maintaining the homeostasis of the organism [123]. With respect to the colonization and formation of the microbiome in fish, it is estimated that it starts in the eggs, by the influence of the surrounding water and in the content of the first food, initially giving discrepancies in the identification of native microbial communities, concluding that there are species-specific colonizing bacteria with which it starts [124].

It is now recognized that the predominant phyla in fish are *Proteobacteria*, *Firmicutes*, *Fusobacteria*, and *Actinobacteria* from the highest to lowest abundance, respectively, although of course there are variations due to diet [125].

### 5.1. Salmon

**Figure 6:**
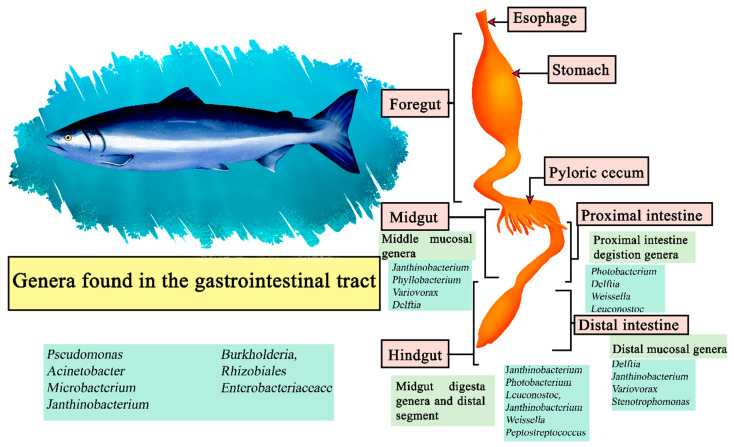
Digestive tract of Atlantic Salmon. Microorganisms frequently found in different sections of the gastrointestinal tract compartments.

The Atlantic Salmon (*Salmo salar*) is carnivorous in nature, with Norway and Chile being its main producers [126]. Its gastrointestinal system is composed of an esophagus, and a U-shaped stomach and intestine with the presence of pyloric caecae attached to it [127]. Although it is a carnivorous fish, it has been proposed to use diets from terrestrial plant sources to improve economic and environmental sustainability, causing farmed Atlantic salmon to change their diet. This has a direct impact on their microbiome, which exhibits variations based on whether their diet is carnivorous or vegetarian [128]. In samples of the digesta in the proximal intestine the most abundant genera were *Photobacterium*, *Delftia*, *Weissella*, and *Leuconostoc*. The genera *Janthinobacterium*, *Photobacterium*, *Leuconostoc*, *Janthinobacterium*, *Weissella*, and *Peptostreptococcus* were the most abundant in the digesta of the midgut and distal segment, and in the middle mucosa *Janthinobacterium*, *Phyllobacterium*, *Variovorax*, and *Delftia*, while *Delftia*, *Janthinobacterium*, *Variovorax* and *Stenotrophomonas* had a higher relative abundance in the distal mucosa. Represented as central microbiota of the salmon were the genera *Pseudomonas*, *Acinetobacter*, *Microbacterium*, *Janthinobacterium*, *Burkholderia*, members of *Rhizobiales*, and *Enterobacteriaceaceace* (Figure 6) [129].

The use of alternative foods such as insects are proposed as potential ingredients for the manufacture of sustainable diets [130]. An experiment developed by Y. Li et al., 2021 used in *Salmo salar* two diets; a commercially relevant reference diet (REF) and produced from black soldier fly (BSF) larvae, which is an important source of chitin. The BSF diet increased the relative abundance of *Actinomyces*, *Bacillus*, *Brevibacterium*, *Corynebacterium*, and *Enterococcus*, highlighting that the microbiota is modified by feed and/or feed composition to the salmon. In addition, the study points out that chitin can favor *Bacillus* bacteria, as they can produce chitinase [131], this being beneficial for the probiotic role of *Bacillus* bacteria against pathogens.

Soybean meal (SBM) has been widely used in fish diets as a vegetable meal to replace fish meal. However, it has limitations for inclusion in carnivorous fish, as it has been shown that inclusion of plant-based feeds can have negative effects on weight gain in salmonids due to anti-nutritional factors [132]. A study conducted in salmon (*Salmo salar*) included two types of diets, the first based on soybean meal (30% SBM) and the second on fermented soybean meal (30% FSBM). The results showed that the *Paracoccus* genus was found in the SMB diet and the *Acinetobacter*, *Shewanella*, and *Altererythrobacter* genera in the FSBM diet. In addition, a higher amount of lactic acid bacteria (LAB), including *Lactobacillus*, *Lactococcus*, and *Pediococcus* of the phylum *Firmicutes*, was reported for both intestinal samples [133].

An experiment conducted by Villasante et al. (2019) used salmon with two experimental diets: a moderate carbohydrate and protein diet (MC/MP; with 15% wheat starch); and a high carbohydrate and protein diet (HC/LP; with 30% wheat starch). The highest to the lowest proportion of phyla found were *Firmicutes*, *Actinobacteria*, and *Proteobacteria*, with 78.6% and 71.8% established for *Firmicutes* in MC/MP and HC/LP diets, respectively. On the other hand, the most relevant genera found were *Planctomycetes* and *Lactococcus* which obtained a significant increase in relative abundance in fish fed the HC/LP diet. This study was able to prove that the ingestion of a diet rich in carbohydrates affects the microbial communities of salmon and, consequently, would benefit carbohydrate-metabolizing bacteria. It is also important to note that the HC/LP diet reported lower weight gain and a daily growth coefficient compared to fish fed the MC/MP diet [134].

### 5.2. Rainbow Trout

**Figure 7:**
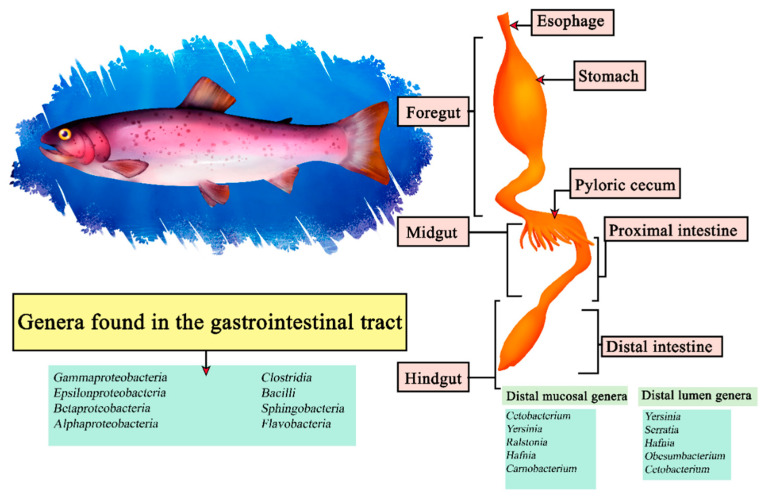
Digestive tract of rainbow trout. Microorganisms frequently found in different sections of the gastrointestinal tract compartments.

The rainbow trout (*Oncorhynchus mykiss*) is a carnivorous fish of the freshwater Salmonidae family, noted for its reproductive efficiency and intestinal tract adaptable to feeding conditions [135]. It belongs to the Teleosts, so it shares characteristics in its intestinal morphology with other fish, presenting a stomach consisting of cardiac, fundic and pyloric regions, forming a J-shape, which by surface extension benefits enzymatic digestion [136]. It has been reported through sequencing *that Cetobacterium, Yersinia, Ralstonia, Hafnia* and *Carnobacterium* are the most abundant within the distal intestinal mucosa and *Yersinia, Serratia, Hafnia, Obesumbacterium* and *Cetobacterium* in the distal lumen [137]. In turn, other studies indicate that under a diet with insect protein directly related genera are *Oceanobacillus, Bacillus, Paenibacillus* and *Cetobacterium* within the intestines [138]. In the case of a central microbiota in *Oncorhynchus mykiss* it is difficult to describe since they encompass around 52 taxa in some studies, from among them we can highlight the classes *Gammaproteobacteria, Epsilonproteobacteria, Betaproteobacteria, Alphaproteobacteria, Clostridia, Bacilli, Sphingobacteria* and *Flavobacteria* (Figure 7) [139].

Several studies have used insects as dietary components in farmed fish due to the nutritional richness they provide in terms of proteins, amino acids, fatty acids, vitamins, and minerals [140]. A study by Terova et al., 2019 also employed the species *Hermetia illucens*, known as black soldier fly, to study how the intake of *Hermetia illucens* (Hi) meal in different proportions: (0% (Hi 0); 10% (Hi 10); 20% (Hi 20); and 30% (Hi 30) can modulate the gut microbiota of rainbow trout (*Oncorhynchus mykiss*). The results indicated the presence of the phyla *Actinobacteria*, *Firmicutes*, and *Proteobacteria*, of which the most influenced by diet were *Actinobacteria* and *Proteobacteria*, which responded better to a higher proportion of Hi, except for *Proteobacteria*, which was decreased in the 20% and 30% treatments. In addition, the order of *Lactobacillales*, which at the genus level includes *Facklamia*, *Enterococcus*, *Lactobacillus*, and *Pediococcus* known as lactic acid bacteria (LAB), was favored in the Hi-containing diets. The genera *Actinomyces*, *Brevibacterium*, *Corynebacterium*, *Leucobacter*, and *Staphylococcus* were also reported. Finally, *Corynebacterium* variabile, *Lactobacillus plantarum*, *Lactobacillus zeae*, *Weissella cibaria*, *Clostridium butyricum*, and *Clostridium fimentarium* were named at the species level in the diets with Hi. Consequently, the experimental diets increased BAL bacteria and butyrate-producing bacteria, and fermentable chitin was stated to be an element acting as a prebiotic [141].

On the other hand, to replace fishmeal (FM) to achieve sustainable aquaculture. Mikołajczak et al. (2020) proposed using insects equally in sea trout (*Salmo trutta*) with: a control diet (CON); 10% hydrolyzed meal of mealworm (*Tenebrio molitor*) (TMD); and a third treatment using the previous proportion in hydrolyzed meal of super worm (*Zophobas morio*) (ZMD). The results indicated that the diet with TMD significantly reduced the concentration of the *Lactobacillus* group and *Carnobacterium* spp. In contrast, the ZMD diet reduced the populations of *Aeromonas* spp., *Enterococcus* spp., and *Carnobacterium* spp. It was concluded that diets with TMD and ZMD play a key role in the health of the microbiota [142].

Under the same previous objective, a method to replace the use of animal proteins from the sea, the use of vegetable proteins was proposed. However, vegetable proteins (VM) have nutritional disadvantages, as they contain non-digestible complex carbohydrates, and a lack of essential amino acids and n-3 polyunsaturated fatty acids (n-3 PUFA). Inclusion of plant protein in fish has presented challenges in providing essential amino acids, also relying on anti-nutritional factors that decrease feed efficiency [143]. Under this context, a study used rainbow trout (*Oncorhynchus mykiss*) with treatments that included: vegetable protein as the control (CV); a fish base diet (CF); and diets containing protein from *Hermetia illucens* (H), known as black soldier fly, where 10% (H10), 30 (H30) and 60% (H60) of the vegetable protein was replaced by C. Also, poultry by-product meal (P) diets were used as another source of animal protein. That description is elaborated from parts extracted from slaughtered poultry being an economical feed, rich in nutrients and essential amino acids, which has been tested in species such as *Sparus aurata* [144]. P was used at 30% (P30) and 60% (P60) of the CV diet. Finally, a combination of 10% and 50% of H and P, respectively, (H10P50) was used. In all diets at the phylum level the populations present were *Proteobacteria*, *Firmicutes*, and *Tenericutes*. With respect to the control, the diets with insect meal had a higher amount of *Gammaproteobacteria*, *Enterococcus*, and *Actinomyces* followed by a decrease in *Pseudomonas*. In addition, the occurrence of *Erysipelothrix* was reported compared to diets with CV or P. Therefore, it is concluded in this study that none of the diets had any negative effect. Diets that included insect meal helped to increase the diversity of intestinal microorganisms, with chitin being the prebiotic agent provided [145]. In summary, diets based on insect meal were beneficial and serve as a replacement for other types of animal protein, obtaining similar nutritional benefits in addition to providing a healthier microbiome.

As for medicinal herbs, Chinese yam (*Dioscorea oppositifolia* L.) is an important traditional Chinese medicinal herb widely cultivated in China and is used in vertebrates to help promote digestion, immunomodulation, antioxidant defense, and as an anti-inflammatory [146]. Its use in rainbow trout diets has not been studied and that is why the team of Wang et al., 2020, designed diets for trout containing: yam extract; a control (CON); and the other 0.1% (O1); 0.2% (O2); and 0.4% (O3) of extract. At the phylum level, mainly *Bacteroidetes*, *Tenericutes*, *Firmicutes*, and *Actinobacteria* were found; for the O1 treatment the population of *Actinobacteria* and *Firmicutes* decreased compared to the control. The opposite happened to the populations of *Patescibacteria*, which increased in the O3 treatment. At the genus level, *Anaeroplasma*, *Candidatus_Saccharimonas*, *Ruminococcaceae*, *Lactobacillus*, *Rikenella*, *Lachnospiraceae*, *Bifidobacterium* and *Marvinbryantia* were mentioned for a total of 10 phyla. Taking into account the changes between phylum populations, it was determined that *Ruminococcoccaceae_UCG*, *Lactobacillus*, *Rikenella*, *Lachnospiraceae* _NK4A136_group, *Eubacterium*, *Bifidobacterium* and *Marvinbryantia* had a lower relative population for the treatment with 0.1% yam extract, while the *Lachnospiraceae* _NK4A136_group and the [Eubacterium]_coprostanoligenes_group were reduced in the treatment with 0.2% extract. On the contrary, in the treatment with 0.4 extract *Bifidobacterium*, *Marvinbryantia*, and *Candidatus_Saccharimonas* increase. It is concluded that at the phylum level the treatment that produced more significant changes was O1 with 0.1% of yam extract, which greatly reduced the microbial diversity, fortunately this was recovered by increasing the concentration in the treatment with 0.4% of extract where at the genus level *Bifidobacterium*, *Marvinbryantia*, and *Candidatus_Saccharimonas* were favored and increased their population. Therefore, *Bifidobacterium*, *Marvinbryantia*, and *Candidatus_Saccharimonas* are beneficial bacteria that regulate homeostasis in the microflora, regulate metabolism and fulfill bactericidal function, treatment with 0.4% of yam extract being the recommended dose [147].

### 5.3. Tilapia

**Figure 8:**
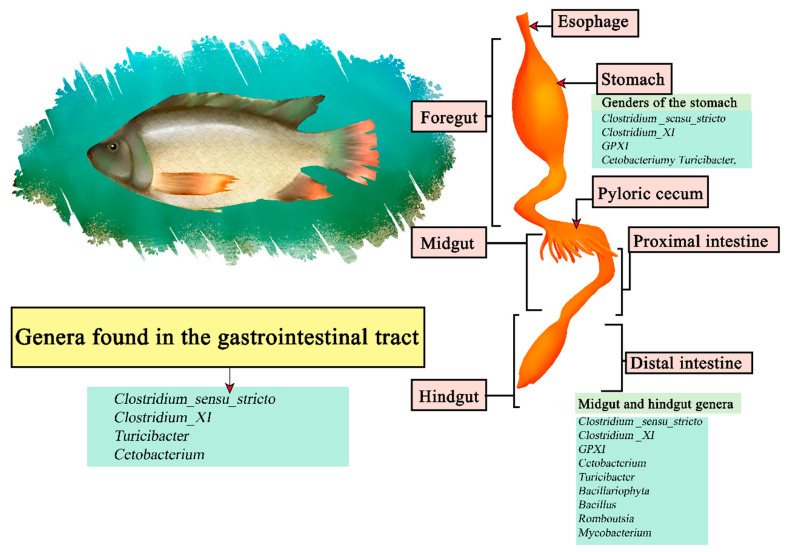
Digestive tract of Nile tilapia. Microorganisms frequently found in different sections of the gastrointestinal tract compartments.

Nile tilapia (*Oreochromis niloticus*) is a freshwater fish native to Africa and the Middle East, being the second most cultured fish in the world [148]. Is omnivorous and with high plasticity focusing on phytoplankton, zooplankton detritus, and macrophytes [149]. It is part of the teleosts, consisting of a gastrointestinal gut consisting of the esophagus, Y-shaped stomach, and intestine [150]. It has been noted that Proteobacteria and Fusobacteria were the central phyla of intestinal microorganisms in tilapia, the most common genus being Cetobacterium within the inner, mid and hindgut [151]. Other studies focused on studying the stomach, midgut and hindgut segments, with sequenced luminal and mucosal samples, determining that the stomach section contains the genera *Clostridium _sensu_stricto*, *Clostridium_XI*, *GPXI*, *Cetobacterium*, and *Turicibacter*. The midgut region *Clostridium _sensu_stricto*, *Clostridium _XI*, *GPXI*, *Cetobacterium*, *Turicibacterium*, *Cetobacterium*, *Cetobacterium*, and *Turicibacter*, *Cetobacterium*, *Turicibacter*, *Bacillariophyta*, *Bacillus*, *Romboutsia*, and *Mycobacterium*. The hindgut *Clostridium _sensu_stricto*, *Clostridium _XI*, *GPXI*, *Cetobacterium*, *Turicibacter*, *Bacillariophyta*, *Bacillus*, *Romboutsia*, and *Mycobacterium*. The results indicate that *Clostridium_sensu_stricto*, *Clostridium_XI*, *Turicibacter*, and *Cetobacterium* are the core genera (Figure 8) [152].

Garlic (*Allium sativum*) possesses antimicrobial properties, and it is reported that its use in fish can help improve growth, survival and provide better resistance to infections (Chen, J. et al., 2021). Garlic has been applied in several studies showing favorable results for *Perca fluviatilis* and *Salmo caspius* species [153,154]. Frequent streptococcal infection in fish leads to economic losses in the aquaculture industry, which is why there is a desire to find solutions to this infection [155]. Tilapia (*Oreochromis niloticus*) is reported to be the fish most susceptible to *Streptococcus iniae* (*S*. *iniae*) infection. In an investigation by Foysal et al., 2019, diets containing 0.5 g and 1.0 g/100 g of commercial garlic powder (TG1 and TG2, respectively) where fed. Fish were used that had been previously injected with bacterial suspension and placed in eight different aquaria: two for control without *S*. *iniae* (C1); and two for control with *S*. *iniae*, but without garlic supplementation (C2S). The last four were used for supplementation of the treatments with garlic and *S*. *iniae* in the mentioned proportions. In the control diets without bacteria, the following populations were found at the phylum level: *Fusobacteria*, *Fusobacteriia*, *Fusobacteriales*, *Leptotrichiaceae*, and *Hypnocyclus*. For the infected control treatments, *Firmicutes*, *Clostridia*, *Proteobacteria*, *Alphaproteobacteria*, *Gammaproteobacteria*, and *Aeromonadales* were found. The most remarkable result included the decrease in OTU counts for Vibrio (pathogen) with the 1.0 g garlic diet. Therefore, it was concluded that supplementation with 1.0 g garlic (TG2) was the most effective against *S*. *iniae* infection by improving the health status of fish [156].

A study by Li et al. (2019) focused on the use of probiotics, a necessary additive for the challenges faced in current aquaculture and the frequent use of antibiotics that induce resistance. The probiotic used was *Clostridium butyricum*, a typical butyric acid bacterium, which can help inhibit the proliferation of pathogenic bacteria. The diets were prepared at different concentrations of the bacterial suspension together with the basal feed: for the control group its concentration was 0 and the other treatments were at concentrations 1 × 10^4^, 1 × 10^5^, 1 × 10^6^ and 1 × 10^7^ CFU g^−1^ of diet (indicated as CG, CB1, CB2, CB3 and CB4, respectively). The diet was applied in tilapia (*Oreochromis niloticus*) and the results indicated 49 phyla, 108 classes, 210 orders, 394 families and 704 genera. At the phylum level, the relative abundance of *Bacteroidetes*, *Firmicutes*, Candidate-division-SR1, *Chloroflexi*, *Chlorobi*, *Acidobacteria*, *Spirochaetae*, *Nitrospirae*, *Parcubacteria*, *Planctomycetes*, and WCHB1-60 increased with respect to the control. Highlighted at the genus level was an increase in the relative abundance of *Cetobacterium*, CKC4, *Aeromonas*, and *Gammaproteobacteria*, in contrast to the CG group. The most interesting results show that the group with the most significant changes was CB2 showing at the phylum level a decrease in relative abundance of *Fusobacterium* and CKC4, and at the genus level Candidate_division_SR1_norank, *Bacteroidetes*_vadinHA17_norank, *Dechloromonas*, *Zoogloea*, *Oligoflexales*_noran, *Comamonadaceae*_unclassified, *Draconibacteriaceae*_uncultured, *Saprospiraceae*_uncultured, *Nitrospira*, *Bacillus*, and WCHB1-69_norank were more abundant. Finally, the authors pointed out that the diet with the most optimal concentration is CB2 (1 × 10^5^ CFU g^−1^), which improves immunity by favoring higher diversity in microbiota and achieves better growth performance [157].

To improve the efficiency of fish feeding, Hassaan et al., 2020, proposed to use inert clays as a supplement for sustainable aquaculture. Sericite is described as a fine white powder of muscovite form, which like other clay materials was used in the practice of geophagy as a gastrointestinal lining against inflammations, and ascribed an antimicrobial role [158]. Nile tilapia were used with a total of five isonitrogenous (315.65 g/kg crude protein) and isocaloric (18.7 MJ/kg crude energy) experimental diets. One control diet was used without sericite supplementation and the other four were fed with different concentrations of sericite (2.5, 5, 7.5, and 10 g/kg). Experimental feeding in tilapia had an effect on the linear growth of bacteria, *E*. *coli* and *Enterobacteriaceae* in the stomach and intestine as sericite supplementation increased, implying that sericite administration may have bactericidal effects to buffer the aqueous pH and oxidation state. At a general level the experimental diet improved growth performance, digestive enzymes, and hematological and immunological parameters in fish [159].

## 6. An Approach to the Functions of Microorganisms

Within the microbiome of production animals, the microorganisms with the highest participation evaluated in this review and assessed by its authors involve at the phylum level the *Bacteroidetes*, which are found in large numbers and represent more than half of the intestinal microbiome of animals. These are in greater numbers in the distal intestine, performing fermentation processes in polysaccharides and producing short-chain fatty acids (SCFA), which can contribute up to 10% of daily calories when the diet is rich in fiber [160]. As ruminants are similar both morphologically and functionally, many of the microorganisms found are coincident in their digestive tract. One genus frequently observed in studies was *Prevotella* belonging to the phylum *Bacteroidetes*. It has been shown that it can prevent rumen acidosis in cattle by preventing the proliferation of acid-producing bacteria that disrupt digestive processes in cattle [161]. On the other hand, its colonization has been shown to decrease the relative abundance of *Firmicutes* such as the *Ruminococcaceae* family known for their fibrolytic activity [162,163]. Although *Prevotella* is present in many animals, its abundance has been increased in diets containing carbohydrates with a high level of dietary fiber as in studies in ruminants [164]. such as Holstein dairy cows and Hu lambs, as proposed by Wang et al. (2020) and Du et al. (2022), respectively. However, the diet proposed by Cremonesi et al. (2018) presented a decrease in the *Prevotella* genus, in the face of a diet with flaxseed as a dietary lipid supplement in alpine goats. This has been observed in diets that integrate a high content of polyunsaturated fatty acids (PUFA), impairing the colonization of the phylum *Bacteroidetes* in ruminants [165].

The phylum that continues in abundance is Firmicutes, gram-negative bacteria associated with the phylum Bacteroidetes, found in similar proportions. Moreover, their interaction is closely related to the performance of anaerobic digestion processes [166]. *Firmicutes* bacteria have been found to be associated with protein and fat metabolic activity. Therefore, a diet with a high amount of protein and fat increases the proportion of *Firmicutes* bacteria [167]. In ruminant animals, bacteria of the species *Ruminococcus flavefaciens* belonging to the phylum *Firmicutes* are cellulolytic bacteria involved in the breakdown of cellulose in the rumen. This gram-positive bacterium is able to form a cellulosome, i.e., a multienzyme complex composed of four protein components (ScaA, ScaB ScaC and ScaE) and cellulose-substrate-degrading enzymes [168,169]. Another cellulolytic bacterium that hydrolyzes polysaccharides in ruminants is *Ruminococcus albus* [21]. As expected, both *Ruminococcus flavefaciens* and *Ruminococcus albus* were found in diets containing fiber in studies described by Zhu et al., 2017 and Yusuf et al., 2017. Now, their decrease in the diet that included *Piper sarmentosum* described by Zhou et al., 2020, hints that bioactive compounds inhibit bacteria of the genus *Ruminococcus*, which has also been described by other authors who describe compounds such as tannins and essential oils exerting an antimicrobial role on gram-positive bacteria [170]. The bacterium *Butyrivibrio fibrisolvens*, present in the gastrointestinal tract of ruminants [171], showed an increase in its population under a diet containing flaxseed oil and vitamin E, described by Yoshimura et al., 2018, due to its role in the biohydrogenation process and the increase in compounds involved in biohydrogenation such as stearic acid (C18:0). As for the genus *Bifidobacterium*, of the phylum *Actinobacteria*, the diet that employed grape pomace in lambs [65], a food with high antioxidant power, presented an increase in its population, a result that agrees with other diets that have implemented antioxidants and that have presented an improvement in meat quality in terms of fatty acid content in lambs [172]. Bacteria of the genus *Lactococcus*, belonging to the order *Lactobacillales*, are considered within the group of lactic acid producing bacteria (LAB) [173]. Their presence was detected in Holstein Friesian cows in the diet presenting *Ascophyllum nodosum* [151]. The increase triggered in *Lactococcus lactis* and *Lactococcus garvieae* populations could be attributed to the fact that *Ascophyllum nodosum*, brown algae composed of polysaccharides, peptides, and nutritional compounds, can be used by the genus *Lactococcus* for the production of lactic acid.

Other phyla, such as proteobacteria, have been found in lesser proportion in the investigations. They are generally gram-negative and are involved in the microbiota as facultative anaerobes, unlike the other microorganisms considered strict anaerobes. In ruminants, in the phylum of proteobacteria is the genus *Succinivibrio*, which has shown that it can produce propionate in the rumen in the same way as the genus *Prevotella* [174]. Its presence has been attributed to higher feed efficiency, higher milk production and its protein quality in dairy cows [175]. In diets proposed by Cremonesi et al. (2018) that integrated flaxseed in alpine goats, the genus *Succinivibrio* was increased, as was also seen before the integration of essential oils in diets [176].

In the studies evaluated, the presence of the phylum actinobacteria, a group of Gram-positive bacteria with genera such as *Micrococcus*, *Mycobacterium*, *Amycolatopsis*, *Frankia*, and *Streptomyces*, was also described. These bacteria are mainly known for generating bioactive natural products, which have played a role in the prevention of gastrointestinal and systemic diseases, giving them therapeutic use in pharmacological industries and as probiotics [177,178].

Additionally, other phyla were found in a smaller proportion, such as *Fibrobacteres*, *Fusobacterium*, and *Euryarchaeota*. In ruminants, bacteria belonging to the phylum *Fibrobacteres* are cellulolytic bacteria capable of degrading polysaccharides and are considered one of the major fiber degraders at the rumen level. The best-known species is the species *Fibrobacter succinogenes* [179]. In the diet proposed by Zhu et al. (2017), which used the fungus *Saccharomyces cerevisiae*, a positive effect on increasing *Fibrobacter succinogenes* was detected, as has also been seen with other cellulolytic bacteria in diets incorporating the fungus [180]. Similarly, diets used in goats [80,81], in treatments including *Andrographis paniculata* and *Piper sarmentosum*, respectively, showed a population increase in *Fibrobacter succinogenes*. In the former, possibly due to the increase in ruminal pH caused by ruminal bacteria, which subsequently provides benefits [181]. On the other hand, the decrease in *Fibrobacter succinogenes* populations after the inclusion of *Piper sarmentosum* suggests that the inhibitory causes of bioactive compounds, such as tannins, affect other gram-positive bacteria, as well as essential oils, which have been shown to inhibit the relative abundance of *Fibrobacter succinogenes* as with other metagenomic bacteria in the rumen [176].

The animals with only one true stomach have a varied morphology being of very different species from each other and even from different habitats. Starting with chickens, we found the genus *Lactobacillus*, belonging to the order *Lactobacillales*, as well as bacteria of the genus *Lactococcus*; therefore, they are proteolytic and fermenters of sugars for the production of lactic acid, and used as probiotics. The diet used by Abu Hafsa & Ibrahim (2018) that includes grape seeds promoted the colonization of *Lactobacillus* in chicks; the same result occurs in other studies using grapes, benefiting the population increase in *Lactobacillus* [182]. The cause is due to the antioxidant power of polyphenols which promotes the growth of lactic acid bacteria such as *Lactobacillus* and *Bifidobacterium* (Pozuelo et al., 2012). Similar results are observed in diets proposed by Kwak et al., 2021, which used a diet that included dietary soropholipids in chicks, and J. H. Park & Kim, 2020, which used an *Achyranthes japonica* in chicks where it increased the population of *Lactobacillus*.

In animals such as swine, *Prevotella* have been found to be associated with positive traits in feed intake, feed efficiency and weight gain, being able to ferment complex dietary polysaccharides [183]. Diets used by Sun et al., 2020, that used sodium butyrate, showed an increase in the *Prevotella* genus in the cecum, where it is attributed that sodium butyrate could positively modify the mucosal walls of the small intestine decreasing the entry of carbohydrates and proteins to the cecum and increasing the population of bacteroidetes including the *Prevotella* genus. This genus has been described as helping the maturation of the intestinal mucosa by the production of acetate [184]. Similar result has also been seen in chickens upon the inclusion of sodium butyrate (800 mg/kg) benefiting bacteroidetes proliferation [185]. The study by C. Wang et al., 2019, used a basal diet with tributyrin which increased the population of *Lactobacillus*, while other studies report that the use of tributyrin decreases *Lactobacillus* populations by reducing intestinal pH due to the production of butyric acid in the hydrolysis of tributyrin [186].

Studies conducted by Villasante et al. (2019) investigated the effects of different carbohydrates levels in the diet of fish species such as *Salmo salar*, *Oncorhynchus mykiss* and *Oreochromis niloticus*. These studies suggest that higher levels of digestible carbohydrates promote and increase in the relative abundance of *Lactococcus* populations. These finding have been replicated in subsequent studies as well [187].

Terova et al., 2019 used a diet based on black soldier fly in rainbow trout which increased the population of bacteria belonging to the genus *Lactobacillus* and butyrate-producing bacteria such as *Clostridium*. Similar results have been seen in chickens [188] and pigs [189] with diets involving *Hermetia illucens*. In addition, it should be considered that the chitin present in insect meal reduces the abundance of *Proteobacteria* as it is a type of insoluble fiber analogous to cellulose found in vegetables [190].

Finally, in cases where the Vibrio genus and *Streptococcus iniae*, a bacterial pathogen known to be a threat against marine species [191], were reduced in their population in the diet incorporating garlic (*Allium sativum*) by Foysal et al., 2019, in tilapia, this is attributed to the beneficial bioactive qualities found in garlic improving the immune response [192].

## 7. Conclusions

The study of the microbiome is multifactorial in nature. The events that cause variations in the population of microorganisms in the intestinal tract of production animals are diverse and are produced mainly by the type of diet and the chain of reactions it generates. However, factors such as the animal’s life stage, external conditions (climatic or captivity), social behavior, and microorganisms native to the intestinal tract can also affect microbial diversity. From the above studies, it is clear that the proportion of microorganisms varies among the different types of feed and that there is a clear difference between species. Ruminants are the most similar, since their microbial communities are related to the degradation of fiber and carbohydrates in ruminal fermentation, including the presence of methanogenic archaea. Similarly, the other species described have their own microbial populations with variations according to their type of diet, which changes as there is a greater relationship between carbohydrates, fat, and proteins, among others. It is emphasized that a diet that includes antioxidants or probiotics helps to promote microbes that benefit health and inhibit the increase in pathogenic strains. In addition, it has been suggested that certain microbial genera are related to positive aspects for production, as in lambs where the presence of *Succiniclasticum* correlates with the final weight. However, there is a lack of information to explain the role of certain microorganisms in a diet, such as the *Cetobacterium* genus that is inhibited in tilapia by the use of probiotics. In general, many of the studies reviewed do not consider aspects such as pH in the different sections of the gastrointestinal tract, morphological and histological aspects that could be key to a better understanding of how microorganisms interact with each other and with the gastrointestinal environment.

## 8. Future Perspectives

Microbiome-based studies will become more relevant as more knowledge is gained about its functions and existing variables. So far, the main focus of microbiome research in production animals has been to understand its changes under defined feeding strategies and how this influences performance. Understanding how diet components and various supplements affect microbial populations in the gastrointestinal tract of production animals could be very useful, since their modulation would be related to obtaining better quality livestock products, as well as better feed efficiency and resource optimization. The use of insects and sea products as potential foods of nutritional value and as replacements for commonly used dietary components of vegetable origin is highlighted. It is hoped that future studies can enhance their nutritional characteristics, taking into account environmental care, especially in the aquaculture area.

## Data Availability

Not applicable.

## Supplementary Materials

The following are available online at: https://www.mdpi.com/article/10.3390/microorganisms11092219/s1, Table S1: Summary of experimental diets in production animals.
